# Physics-Informed Online Learning for Temperature Prediction in Metal AM

**DOI:** 10.3390/ma17133306

**Published:** 2024-07-04

**Authors:** Pouyan Sajadi, Mostafa Rahmani Dehaghani, Yifan Tang, G. Gary Wang

**Affiliations:** Product Design and Optimization Laboratory, Simon Fraser University, Surrey, BC V3T 0A3, Canada; sps11@sfu.ca (P.S.); mra91@sfu.ca (M.R.D.); yta88@sfu.ca (Y.T.)

**Keywords:** physics-informed neural networks, metal additive manufacturing, online learning, real-time modeling, temperature field prediction

## Abstract

In metal additive manufacturing (AM), precise temperature field prediction is crucial for process monitoring, automation, control, and optimization. Traditional methods, primarily offline and data-driven, struggle with adapting to real-time changes and new process scenarios, which limits their applicability for effective AM process control. To address these challenges, this paper introduces the first physics-informed (PI) online learning framework specifically designed for temperature prediction in metal AM. Utilizing a physics-informed neural network (PINN), this framework integrates a neural network architecture with physics-informed inputs and loss functions. Pretrained on a known process to establish a baseline, the PINN transitions to an online learning phase, dynamically updating its weights in response to new, unseen data. This adaptation allows the model to continuously refine its predictions in real-time. By integrating physics-informed components, the PINN leverages prior knowledge about the manufacturing processes, enabling rapid adjustments to process parameters, geometries, deposition patterns, and materials. Empirical results confirm the robust performance of this PI online learning framework in accurately predicting temperature fields for unseen processes across various conditions. It notably surpasses traditional data-driven models, especially in critical areas like the Heat Affected Zone (HAZ) and melt pool. The PINN’s use of physical laws and prior knowledge not only provides a significant advantage over conventional models but also ensures more accurate predictions under diverse conditions. Furthermore, our analysis of key hyperparameters—the learning rate and batch size of the online learning phase—highlights their roles in optimizing the learning process and enhancing the framework’s overall effectiveness. This approach demonstrates significant potential to improve the online control and optimization of metal AM processes.

## 1. Introduction

Metal additive manufacturing (AM) introduces a paradigm shift in the field of manufacturing technologies, significantly enhancing adaptability across a range of sectors such as aerospace, biomedical engineering, and defense [1]. The capacity of this technology to construct detailed, tailor-made 3D configurations through sequential layer deposition not only enables the realization of mass customization but also facilitates the fabrication of components with reduced mass, optimized material consumption, and expedited prototype development [2].

Central to understanding and optimizing metal AM processes is the Parameter–Signature–Quality (PSQ) model, which explains the complex relationships between the settings of the manufacturing process (Parameters), the observable effects during the process (Signatures), and the characteristics of the final product (Qualities). The PSQ model serves as a critical framework, clarifying how variations in process parameters influence the physical and mechanical properties of the final product through changes in process signatures [3,4,5]. This relationship is key to ensuring that the manufacturing process yields parts that conform to predefined quality and performance standards, allowing for precise control over the variables to achieve the desired outcomes [6].

Temperature emerges as a vital signature in this framework, with its management and prediction being fundamental to the integrity and quality of AM parts [7,8]. In metal AM, the rapid heating and cooling cycles lead to significant temperature fluctuations within the substrate and deposited layers, affecting part quality and integrity through stress, distortion, and microstructural changes [9,10]. Temperature variations can also cause defects such as porosity due to trapped gas bubbles, a lack of fusion from insufficient melting, and surface rugosity from thermal gradients. Non-uniform cooling rates can lead to heterogeneous microstructures and low ductility, compromising mechanical properties. Additionally, trapped inert gas bubbles can form due to improper shielding gas flow or high scanning speeds, further degrading part quality. These defects have been widely reported in the literature [11,12,13,14]. In addressing these challenges, real-time or near-real-time temperature prediction models are essential. They enable the dynamic adjustment of process parameters based on thermal feedback, thus enhancing thermal management and reducing defects for improved precision and quality of the final product [15].

Therefore, the capability to monitor and control temperature directly influences PSQ dynamics, impacting thermal signatures and, consequently, the structural and material properties of manufactured components. In improving control over the AM process, these developments not only fulfill specific application requirements but also push the limits of manufacturing efficiency and product innovation in AM.

There has been extensive study on offline, data-driven approaches for thermal modeling in metal AM. Offline, or batch learning, is a method where models are trained on a complete dataset before deployment, without updating or learning from new data during operation. These studies have explored various methodologies for predicting temperature distributions and their effects on the final product’s quality, relying on historical data and computational simulations to inform their predictions. For instance, research by Pham et al. [16] developed a feed-forward neural network surrogate model to accurately predict temperature evolution and melting pool sizes in metal bulk samples fabricated using the directed energy deposition (DED) process. In another work, Mozaffar et al. [17] developed a recurrent neural network (RNN)-based model to predict the thermal history of manufactured parts. Utilizing a considerable amount of data produced by the Finite Element Method (FEM), this model is adept at forecasting temperature fields both on the surface and within the interior of fabricated parts. Moreover, in [18], Le et al. used FEM data from five processes with different currents and voltages to train a neural network for temperature prediction at mesh points, using coordinates, travel speed, and current as inputs. The model, demonstrating over 99% accuracy, can predict temperature histories in new cases.

Adding to these conventional approaches, physics-informed neural networks (PINNs), as introduced by Raissi et al. [19], have emerged as a novel machine learning paradigm by integrating physical laws, typically described by partial differential equations (PDEs), directly into the neural network architecture. Notable implementations include the study by Zhu et al. [20], which utilized PINNs for temperature and melt pool dynamics in Laser Powder Bed Fusion (LPBF), and the research by Xie et al. [21], focusing on temperature prediction in DED. Additionally, Jiang et al. [22] demonstrated the effectiveness of PINNs in melt pool temperature predictions with limited training data. These instances underscore the potential of PINNs to improve predictive accuracy and computational efficiency in metal AM by employing physical principles, especially when faced with sparse data.

However, both traditional offline models and PINNs face shortcomings that limit their practicality within the evolving domain of metal AM. A shared challenge is their limited adaptability to real-time manufacturing variations, frequently leading to discrepancies between predicted outcomes and the dynamics of actual processes. These approaches struggle to generalize across the diverse AM processes characterized by different materials, geometries, and process parameters. Additionally, data-driven models require large training datasets, which are often not available in manufacturing. Meanwhile, PINNs, despite leveraging physical laws for prediction, typically focus on narrow segments of the AM process, which curtails their overall utility. Lastly, these models often require extensive computational resources and processing time for training, which can impede swift decision making critical for optimizing AM processes in real time. For example, the processing time reported in [17] reached 40 h, rendering it impractical for scenarios requiring real-time control.

Other approaches, such as analytical or numerical methods, can address some of these limitations but still have their challenges. Recently, Yang et al. [23] implemented an analytical model with three different heat sources to predict melt pool dimensions and temperature distributions. Their study effectively analyzed the effects of laser processing parameters and material thermophysical properties on the temperature field and melt pool size, offering valuable insights into the intricate thermal behavior in metal additive manufacturing. However, analytical models lack real-time adaptability and cannot incorporate dynamic process variations.

Online learning, a branch of machine learning, updates model parameters dynamically with the arrival of new data, presenting a flexible alternative to conventional offline approaches. This method ensures that the model stays relevant and accurate through real-time adaptation and is more efficient due to its reduced memory storage needs [24]. Unlike batch learning, which requires access to the entire dataset for training, online learning processes data incrementally, removing the need for substantial data storage. The benefits of online learning are evident across various fields. For example, Yang et al. [25] designed an online deep learning model for the continuous monitoring of train traction motor temperatures, adjusting the model’s structure in response to new data. Wang et al. [26] proposed a combined method that leverages offline learning’s predictive capabilities with online learning’s adaptability for the real-time control of deformable objects.

In the realm of metal AM, the application of online learning is emerging. Ouidadi et al. [27] applied online learning for real-time defect detection in Laser Metal Deposition (LMD), using transfer learning and adaptive models like K-means and self-organizing maps to enhance quality control by updating predictions with incoming data. Mu et al. [28] developed an online simulation model for Wire Arc Additive Manufacturing (WAAM) that employs neural network techniques to adapt predictions based on live data, showcasing an improvement over conventional models. Despite these advances, a significant gap exists in the specific application of online learning for thermal modeling within metal AM. In one of the first attempts at surrogate modeling in this context, our recent study [29] proposed an online thermal field prediction method using artificial neural networks for thermal field mapping and a reduced-order model (ROM) for thermal field reconstruction, demonstrating its effectiveness in various experiments and simulations. However, this study is limited to thin wall structures, which restricts its applicability to more complex geometries in metal AM. Additionally, the method requires multiple pyrometers that need to be moved as new layers are added, increasing the complexity and potential for measurement errors.

To the authors’ best knowledge, this study is the first to explore physics-informed online learning for thermal modeling in metal AM, marking a pioneering step into this research area. Our approach improves upon the existing state-of-the-art approaches by offering dynamic adaptability to real-time data, enhanced predictive accuracy, and greater flexibility and generalizability across different settings. Furthermore, it reduces the need for extensive sensor setups, addressing practical challenges in the field.

To this end, this paper introduces a framework that integrates a physics-informed neural network (PINN) with transfer learning and online learning to address the challenges of thermal modeling in metal AM. At its core, this methodology leverages real-time temperature field data alongside heat boundary conditions to accurately predict 2D temperature fields at future timestamps for processes previously unseen and for which no prior data are available before training. This novel approach is distinguished from prior methods by its dynamic adaptability to a wide array of AM scenarios, including variations in geometries, deposition patterns, and process parameters. This adaptability significantly improves the precision and utility of thermal field modeling in metal AM. Our PINN approach offers several unique advantages, including the following:

1. **Real-Time Adaptability:** The PINN approach can quickly adapt to new data in real time, allowing for immediate updates and predictions during the manufacturing process. This is vital for applications requiring rapid decision making and adjustments.
2. **Faster Computation Times:** Neural networks, once trained, can perform predictions much faster than traditional numerical simulations. This speed advantage is crucial for the real-time monitoring and control of the AM process.
3. **Continuous Learning:** The PINN framework can continuously learn and improve from new data, enhancing its predictive accuracy over time. This capability allows the model to become more robust and reliable with ongoing use.
4. **Utilizing Process Data:** The PINN model leverages data gathered directly from the manufacturing process, allowing for more accurate and context-specific predictions. This use of real-time process data helps tailor the model to the specific conditions of and variations in the ongoing AM process, further enhancing its applicability and precision.

The organization of the rest of this paper is as follows. Section 2 introduces the physics-informed online learning framework tailored for real-time temperature field prediction. Section 3 outlines the data generation and model implementation strategies. Section 4 discusses the results, emphasizing the framework’s capabilities and exploring potential limitations. Finally, Section 5 concludes this paper, summarizing the contributions and suggesting future directions for enhancing the adaptability and precision of thermal modeling in metal AM.

## 2. Methodology

In this section, we delve into the development of the proposed physics-informed online learning framework aimed at the prediction of 2D temperature field in metal AM processes. The framework consists of two distinct phases. Initially, an offline learning phase is conducted, where a PINN, incorporating a neural network with physics-informed modifications, is trained on a dataset from a previously conducted metal AM process. This initial phase enables the PINN to grasp the fundamental patterns and dynamics inherent in AM thermal processes, creating a robust foundation of knowledge for subsequent real-time data exposure.

In the online learning phase, this pre-trained PINN serves as the base model for dynamic adaptation to an unseen process, for which data are acquired in real time. The integration of Synaptic Intelligence (SI) [30] and an adaptive learning rate helps maintain and apply previously acquired knowledge as the PINN adapts to new data from different AM processes. This setup enables the PINN to dynamically update its weights through online gradient descent [31] in response to new information, demonstrating its flexibility and prompt response to temperature changes in the new process.

**Figure 1:**
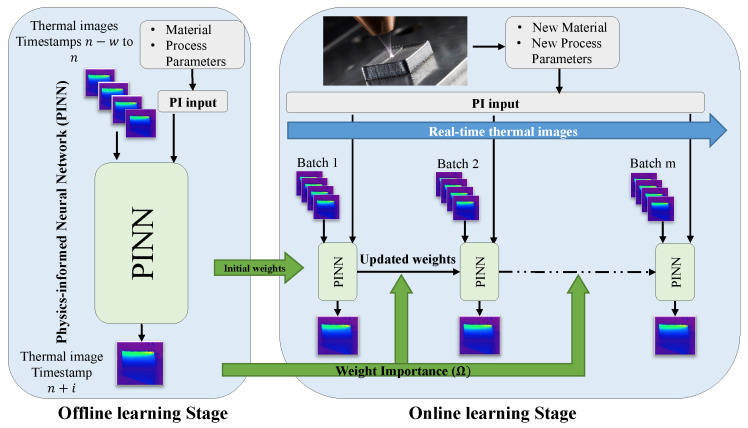
Schematic of the two-stage framework for online 2D temperature prediction in metal AM. Both stages leverage physics-informed components based on process parameters and material to guide the PINN: initially through training on previous AM thermal patterns, and subsequently through updates with real-time thermal images.

The PINN’s architecture consists of three core components: the neural network, physics-informed (PI) input, and a PI loss function. It employs a series of thermal images to predict the 2D temperature field at future time steps, which capture the 2D temperature fields of the currently deposited layer of the manufactured part, along with a PI input that includes heat input characteristics. More precisely, the model inputs a sequence of *w* thermal images spanning from timestamps (n−w) to (n) and the process’ heat input characteristics. It uses these data to forecast the thermal image (i.e., 2D temperature field) at the timestamp (n+i). Here, *w* represents the window size of input data, capturing a specific range of thermal imaging, and *i* denotes the hyperparameter indicating the future timestamp targeted for prediction, focusing on the evolving thermal conditions of the currently deposited layer. Figure 1 presents an overview of the proposed framework.

It is worth mentioning that while thermal imaging provides valuable temperature distribution data, its absolute values can be unreliable due to the non-linearity of gray body emissions in high-temperature metals. This challenge can be mitigated by incorporating a pyrometer for calibration or using two synchronized sensing devices at different wavelengths to calculate temperature based on emissions using Planck’s Law [32]. Additionally, hyperspectral thermal imaging and two-wavelength pyrometry can offer accurate temperature distributions without needing emissivity adjustments [33].

We will further elaborate on the proposed framework in the following sections: Section 2.1 delves into the components of the PINN; Section 2.2 discusses the pretraining phase, highlighting how the model is prepared using historical AM data; finally, Section 2.3 covers the online learning phase, detailing the adaptation of the pre-trained PINN to new, real-time AM processes.

### 2.1. Proposed Physics-Informed Neural Network

**Figure 2:**
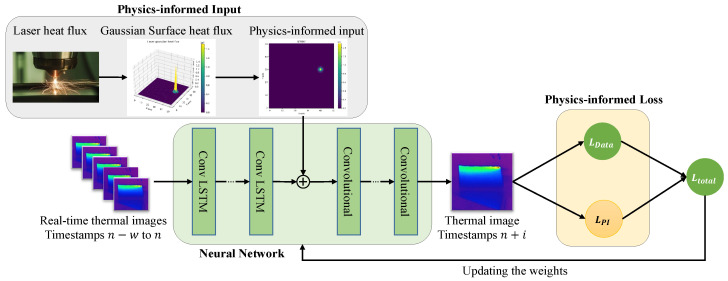
PINN with its components: the neural network, PI input, and PI loss.

The PINN used in our framework is designed to integrate real-time data with physics-based constraints, enabling accurate predictions of thermal fields in metal additive manufacturing This specialized neural network incorporates convolutional long short-term memory (ConvLSTM) layers and convolutional layers into the model’s architecture. It also includes an auxiliary input for process heat flux information, represented as a 2D matrix (i.e., PI input), and integrates a boundary condition (BC) loss term into the overall loss function (i.e., PI loss). Each component is essential for accurately predicting the thermal field by leveraging both data-driven insights and physics-informed constraints. In Figure 2, the proposed PINN—which comprises three key components, the neural network, PI loss, and PI input—is presented.

#### 2.1.1. Neural Network Architecture

The architecture of the neural network is designed to capture the complexities of thermal field prediction within the domain of metal additive manufacturing. Central to this architecture are the convolutional long short-term memory (ConvLSTM) layers, which can simultaneously process spatial and temporal information, making them an ideal choice for tasks addressed in this paper, where 2D thermal images serve as inputs, and the goal is to predict a 2D temperature distribution evolving over time. The choice of CNNs and RNNs over ANNs is due to the need to handle both spatial and temporal dependencies in image data, which is critical for accurate thermal field prediction. To complement the ConvLSTM layers, the architecture also includes traditional convolutional layers. These layers specialize in extracting spatial features from each thermal image, identifying intricate patterns and temperature distributions essential for constructing an accurate predictive model. This architecture is superior because it leverages the strengths of both CNNs for spatial feature extraction and RNNs for temporal sequence modeling, leading to more precise and reliable predictions. Together, these neural network components form a powerful and efficient architecture designed to tackle the challenges of predicting thermal fields in the dynamic environment of metal AM processes.

#### 2.1.2. Physics-Informed Input

In our framework, the Physics-Informed (PI) input is incorporated to infuse the model with physics-based information regarding the heat input of the manufacturing process, thereby enabling the model to capture the intricate relationship between process parameters and the resultant temperature field. This addition ensures that the neural network can access a richer set of data that reflect the underlying physical processes, enhancing its ability to make better predictions. We chose the laser heat flux as the PI input. This parameter serves as a quantifiable measure of the energy intensity and distribution as the laser interacts with the material, which is vital for understanding the thermal dynamics involved in the process. The laser heat flux represents a critical factor that influences key thermal phenomena, including the melting and solidification processes and the creation of thermal gradients within the layer currently under fabrication.

The process of accurately estimating the laser heat flux is a cornerstone in enhancing our model’s predictive accuracy. In the literature, laser heat flux is modeled in different modes, namely point heat source, Gaussian surface heat source, and Gaussian body heat source [23]. Building on this foundation, we utilized a Gaussian surface heat flux model [34] to facilitate a nuanced representation of how the laser’s energy is dispersed across the material layer. This model incorporates critical factors such as laser power (*P*), the radius of the laser beam ($r_{\mathrm{beam}}$), and the material’s absorptivity (η) to create a comprehensive picture of the energy input:

(1) $$q_{\mathrm{laser}}\left(x,y\right)=-\frac{2\eta P}{\pi r_{\mathrm{beam}}^{2}}\exp\left(\frac{2d^{2}}{r_{\mathrm{beam}}^{2}}\right)$$

**Figure 3:**
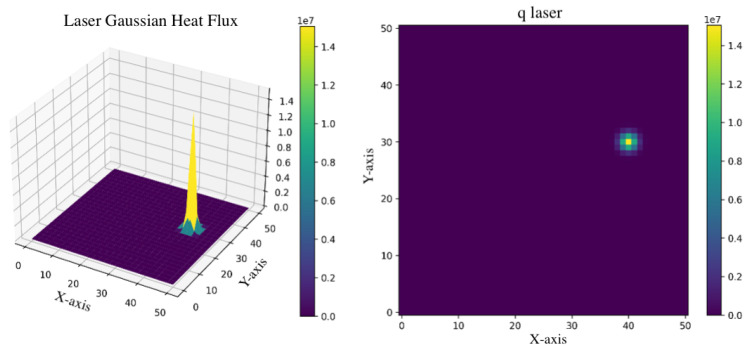
An example of Gaussian surface heat flux on a surface (**left**) and its corresponding $q_{laser}$ matrix (**right**).

This equation calculates the heat flux (i.e., $q_{laser}$) at each point (x,y) on the material’s surface, based on the distance *d* from the laser center, effectively mapping out the spatial energy profile imposed by the laser. The precision in capturing this energy distribution is critical for simulating the thermal dynamics during the additive manufacturing process accurately. It allows our model to predict the resulting temperature fields with sufficient fidelity, considering how variations in laser settings or material properties could impact the thermal environment within the layer being printed. Figure 3 illustrates an instance of laser application on a surface, modeled using the Gaussian surface heat flux, along with its corresponding $q_{laser}$ matrix.

#### 2.1.3. Physics-Informed Loss

In our PINN, the PI loss function is essential to reflecting the physical laws governing the system. The PI loss is designed to specifically enforce boundary conditions, which are critical for accurately modeling thermal processes in metal additive manufacturing.

**Figure 4:**
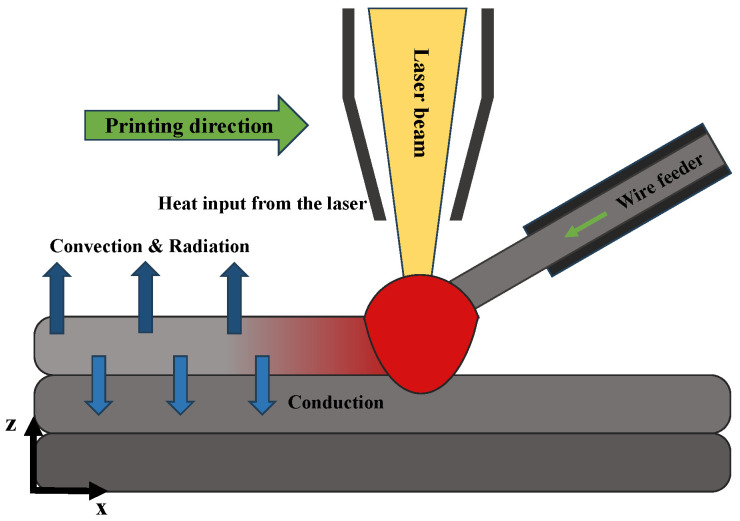
The boundary conditions used in the physics-informed loss function for a DED process, illustrating the heat transfer mechanisms, including heat from the laser, heat conduction to the layer below, convective heat loss, and radiative heat loss.

Boundary conditions dictate how surfaces of a part interact with their environment, influencing heat transfer mechanisms such as conduction, convection, and radiation, which are crucial for predicting temperature fields during manufacturing. Figure 4 showcases the three heat transfer mechanisms considered in our problem. The PI loss minimizes the residual of the physical equations at these boundaries, improving the network’s adherence to physical constraints and ensuring that predictions are both statistically accurate and physically plausible.

By penalizing deviations from these physical laws, our model’s reliability is bolstered. For the problem of predicting the 2D temperature field of the currently deposited layer, the specific boundary condition for the top surface is mathematically expressed as follows:

(2) $$-k\frac{\partial T}{\partial n}=h_{c}\left(T-T_{\mathrm{amb}}\right)+\sigma \varepsilon \left(T^{4}-T_{\mathrm{amb}}^{4}\right)+Q_{\mathrm{laser}}$$

In this equation, *T* denotes the matrix of the 2D temperature fields, representing the temperature distribution during the manufacturing process captured by thermal images. This equation encompasses several heat transfer mechanisms: The term $-k\frac{\partial T}{\partial \vec{n}}$ calculates the conductive heat flux through the surface, with $\vec{n}$ denoting the normal direction outward from the surface. This term accounts for interlayer heat conduction in the normal direction (z-axis or interlayer direction). $h_{c}$ is the convective heat transfer coefficient, modeling heat loss caused by air or fluid motion over the surface. Radiative heat loss is given by $\sigma \epsilon (T^{4}-T_{\mathrm{amb}}^{4})$, where σ is the Stefan–Boltzmann constant, and ϵ is the emissivity, indicating energy lost as radiation based on the fourth power of the temperature difference between the surface and ambient air. $Q_{\mathrm{laser}}$ represents the heat input from the laser, crucial for the melting and fusing of material layers. Figure 4 illustrates the boundary conditions used in the physics-informed loss function. Constants *k*, $h_{c}$, σ, and ϵ are set based on the values for the materials used in the simulation, derived from the material data in the simulation software.

Given the boundary condition, the residual for the boundary condition can be defined as

(3) $$R_{PI}\left(T\right)=k\frac{\partial T}{\partial \vec{n}}+h_{c}\left(T+T_{\mathrm{amb}}\right)+\sigma \epsilon \left(T^{4}-T_{\mathrm{amb}}^{4}\right)+Q_{\mathrm{laser}}$$

The PI loss, $L_{PI}$, is computed as the mean squared error of the residuals across *N* thermal images:

(4) $$L_{PI}=\frac{1}{N}\sum_{i=1}^{N}R_{PI}^{2}\left(T_{i}\right)$$

The total loss function for the PINN, which includes both the data-based loss ($L_{Data}$) and the physics-informed loss ($L_{PI}$), is weighted to balance the contribution from each component. The weights for each loss term, $w_{PI}$ for $L_{PI}$ and $w_{D}$ for $L_{Data}$, are established during the training process. This involves observing and adjusting the impact of each loss term to ensure that they contribute equally to the overall loss. The combined loss function is expressed as follows:

(5) $$L_{Total}=w_{PI}L_{PI}+w_{D}L_{Data}$$

Here, $w_{PI}$ and $w_{D}$ denote the weights assigned to the PI loss and data loss, respectively. These weights are proportionally set to balance the scale between two terms in the loss function, contributing to the improved training robustness of the model.

It is worth mentioning that the physical principle in Equation (2) cannot capture all the complex phenomena involved. The physics incorporated have some limitations. Firstly, it does not account for the intricate nature of heat transfer in the porous material ahead of the heat source, or fully capture the dynamics of the initial layers. Additionally, the assumption of a semi-infinite plate may not accurately reflect the physical realities at the metal powder size resolution. However, our approach is open to incorporating more detailed physics equations, which will strengthen the technology as a general method in future studies.

### 2.2. Offline Learning Stage

In the offline learning stage, our framework trains a PINN on data from a completed metal AM process. This training integrates physics-based inputs and loss functions tailored to the specific material properties and heat input characteristics of the process, equipping the model with an understanding of the underlying thermal dynamics. This comprehensive preparation sets a solid foundation for the model’s subsequent application in the online learning stage.

The PI loss function in our framework incorporates material properties—the thermal conductivity (*k*) and convection heat transfer coefficient ($h_{c}$), determined by the materials used in the process. These properties are crucial for enforcing realistic boundary behaviors for accurate thermal modeling. Additionally, heat input parameters such as laser power (*P*), beam radius ($r_{\mathrm{beam}}$), and material absorptivity (η), are integrated into a physics-informed input that characterizes the laser heat flux.

As the model transitions to the online learning phase, it requires the careful management of model updates to ensure that new data do not drastically alter the neural network’s established weight configurations. It is essential to maintain the network’s fundamental knowledge from the offline learning phase, enabling it to adjust to new data while preserving its accuracy and ability to predict temperature fields as taught by historical data. Synaptic Intelligence addresses this by evaluating the significance of each weight relative to the tasks mastered previously. This evaluation is captured mathematically by omega values (Ω), which highlight weights that are key to the model’s prior tasks and should therefore be conservatively adjusted with incoming data.

(6) $$\Omega_{i}=\frac{\sum_{t}g_{i,t}\Delta w_{i,t}}{{\left(\Delta w_{i}\right)}^{2}}$$

Here, $\Omega_{i}$ is the omega value for weight *i*, $g_{i,t}$ is the gradient of the loss with respect to weight *i* at time *t*, $\Delta w_{i,t}$ is the change in weight *i* at time *t*, and $\Delta w_{i}$ is the total change in weight *i* over the training period. To streamline computation and avoid potential issues like division by zero, an approximation is used:

(7) $$\Omega_{i}\approx \sum_{t}g_{i,t}^{2}$$

This approach posits that the importance of weight is indicated by the sum of its gradients’ magnitudes over time, implying that weights with consistently significant gradients are more important for the model’s function than otherwise.

Incorporating these omega values into the online learning phase allows the model to adjust weight importance flexibly, integrating new data while maintaining the core insights gained previously. The careful balance between incorporating new information and preserving valuable existing knowledge improves the model’s adaptability and performance across varied additive manufacturing scenarios.

### 2.3. Online Learning Stage

In the online learning phase, the primary objective is to predict the temperature field for a new, previously unseen metal AM process that may exhibit characteristics different from the process used during the offline learning stage. This phase is crucial for allowing the model to transition smoothly from utilizing foundational knowledge to integrating new insights, thus maintaining accuracy and adaptability during the dynamic conditions of AM processes. A critical aspect of this phase involves updating the PI input and PI loss function to incorporate new process characteristics as they are encountered. For instance, modifications in laser power, material properties, or beam radius require adjustments to the PI input that captures the laser heat flux and the PI loss terms that enforce adherence to new boundary conditions and material behaviors. By dynamically adapting these PI components to reflect new process characteristics, the model effectively incorporates updated knowledge, enhancing its accuracy and adaptability across different AM setups.

In this phase, the PINN, previously trained during the offline learning stage, serves as the initial model. Its weights are updated in real time as new data from the ongoing process are received. The PINN employs online gradient descent (OGD) to dynamically adapt to new data. This method allows for immediate adjustments to the model’s parameters, enhancing its ability to refine predictions continuously as new information becomes available. The mathematical expression for updating the weights through OGD is shown below:

(8) $$w_{t+1}=w_{t}-\alpha \nabla L\left(w_{t},x_{t},y_{t}\right)$$

where $w_{t}$ and $w_{t+1}$ represent the weights before and after processing the new data point, respectively. The learning rate, α, determines the step size for the update, and $\nabla L(w_{t},x_{t},y_{t})$ is the gradient of the loss function relative to the weights for the current data point $x_{t}$ and its target value $y_{t}$.

The loss function used during this phase includes components for data loss ($L_{\mathrm{data}}$), PI loss ($L_{\mathrm{PI}}$), and Synaptic Intelligence (SI) loss ($L_{\mathrm{SI}}$):

(9) $$L_{\mathrm{total}}=w_{\mathrm{Data}}L_{\mathrm{data}}+w_{\mathrm{PI}}L_{\mathrm{PI}}+w_{\mathrm{SI}}L_{\mathrm{SI}}$$

where $L_{\mathrm{SI}}$ is specifically formulated as

(10) $$L_{\mathrm{SI}}=\sum_{i}\Omega_{i}{\left(\Delta w_{i}\right)}^{2}$$

Here, $w_{\mathrm{Data}}$, $w_{\mathrm{PI}}$, and $w_{\mathrm{SI}}$ are the weights that are determined during the training process to maintain a balanced scale among the various terms in the loss function. $\Omega_{i}$ quantifies the importance of each weight *i* in the neural network, reflecting its role in prior tasks learned by the model. The term $\Delta w_{i}$ measures the change in weight *i* due to new data, and squaring this change ${\left(\Delta w_{i}\right)}^{2}$ aims to minimize large shifts in critical weights, thus preserving essential knowledge and mitigating catastrophic forgetting. This strategic formulation ensures that the model not only adapts to new data but also retains accuracy and stability in predictions by balancing novel learning with the preservation of previously acquired knowledge.

Additionally, we adjust the learning rate dynamically, starting with a lower value to prevent drastic parameter shifts when limited data are available. This conservative approach maintains model stability. As more data are integrated and the model adapts, we increase the learning rate to accelerate learning and enhance adaptability, ensuring that the model remains responsive to new information while preserving its accuracy.

## 3. Data Generation and Model Implementation

In this section, we outline the dataset generation using simulations, emphasizing the distinct characteristics of these simulations. We utilized finite element simulations with ANSYS software, specifically the Workbench AM DED 2022 R2 module, to create the training and testing datasets for our online learning framework. We conducted a total of four simulations, varying materials, geometries, deposition patterns, and process parameters to evaluate the framework’s generalizability under diverse conditions.

These simulations standardized the pass width and layer thickness at a consistent 1 mm. Materials such as 17–4PH stainless steel and Inconel 625 were chosen for their prevalent use and unique properties pertinent to our study. Geometrically, cylinders and cubes were explored for their common industrial applications, and three distinct deposition patterns were investigated to further assess the framework’s flexibility.

For process parameters, we defined two distinct scenarios: one with a deposition speed of 10 mm/s and a higher laser power, and another at 6 mm/s with a lower laser power. Our thermal analysis incorporated factors such as thermal conductivity, convection, and radiation, maintaining substrate and ambient temperatures at a constant 23 °C. Activation temperatures were set at 2000 °C for the lower laser power and 2400 °C for the higher laser power, noting that the simulation abstracts from directly modeling the laser’s heat flux. Our study focused on multi-layer fabrications, where each layer follows the same printing pattern and geometry. This approach was chosen to systematically investigate the thermal behavior and ensure consistency across layers. The number of layers for these processes were 10, 10, 9, and 8. An overview of different simulated processes A–D is illustrated in Table 1.

Although only four simulations were conducted, they were deliberately designed to encompass a range of characteristics, including variations in material types, geometries, deposition patterns, and laser parameters. This diversity was intended to validate the transferability of knowledge from one process to another. The boundary conditions for these simulations were carefully chosen to replicate realistic metal AM processes. Specifically, we considered factors such as thermal conductivity, convection, and radiation, while maintaining constant ambient conditions. Despite these efforts, simulation data cannot fully capture the complexities and variabilities of actual AM processes, such as unforeseen environmental factors and material inconsistencies. Future work should extend the number of simulations and incorporate experimental data to enhance the reliability and applicability of the findings.

Each simulation produced datasets capturing transient temperature values at each timestamp, from which approximately 16,000 input–output pairs per simulation were extracted for training and validation. These pairs consist of sequences of the 2D temperature field for the currently deposited layer as inputs, with the outputs representing the 2D temperature field of the same layer for the subsequent timestamp.

The model was implemented in TensorFlow. We constructed a neural network with six ConvLSTM layers and four convolutional layers, each utilizing 20 filters. This network was pre-trained using data from the simulated processes, with a learning rate set to $10^{-5}$. The pretraining phase employed three previous timestamps (w=3) to predict the temperature field five seconds ahead (i=50). Upon completing pretraining, both the model’s parameters and the omegas for each parameter were saved. These omegas indicate the importance of the weights in retaining learned knowledge, which is vital for the subsequent online learning phase.

In the online learning stage, the architecture remains unchanged, and the pre-trained model, equipped with the saved weights, is introduced to streaming data from a new process simulation.

Based on the insights from Section 4, the learning rate is dynamically adjusted throughout the phase, starting at $5\times 10^{-8}$ and incrementally increasing to $5\times 10^{-5}$ to better accommodate learning from the dynamic, real-time data. While setting $w_{\mathrm{Data}}$ to 1, the regularization parameter $w_{\mathrm{SI}}$ is set to $10^{-5}$ to balance the scales of the data loss ($L_{\mathrm{Data}}$) and the Synaptic Intelligence loss ($L_{\mathrm{SI}}$), ensuring harmony between adapting to new information and preserving essential insights from previous learning. Similarly, $w_{\mathrm{PI}}$ is set to $10^{-15}$ to align the physics-informed loss ($L_{\mathrm{PI}}$) with the other terms in the loss function, maintaining consistency across the model’s evaluation criteria. This calibration supports the neural network to be effectively trained on the fly with the new process data, ensuring the model’s continuous adaptation and robustness in predicting temperature fields across varied additive manufacturing scenarios.

## 4. Results and Discussion

To evaluate the real-time adaptability of our online learning framework to new data, we carried out a sequence of experiments. We designated Process A (Table 1) as the baseline dataset for the offline learning phase, creating a foundational knowledge base for the model to understand the standard thermal patterns of metal AM processes. We then transitioned to the online learning phase, progressively introducing datasets from Processes B, C, and D (Table 1). Each dataset represented a unique scenario and varied incrementally from the parameters of Process A.

The experimental processes were strategically designed to evaluate the performance of the online learning framework under various scenarios: Process B mirrored Process A, differing only in specific operational parameters to test the model’s sensitivity to such changes under controlled conditions. Process C presented a greater challenge by varying not only the process parameters but also the deposition patterns, assessing the model’s adaptability to both thermal and process changes. Process D, introducing a change in geometry and material, represented the most significant departure from the initial setup, testing the model’s ability to adapt predictions to new structural contexts.

To evaluate the model’s performance, we used the Mean Absolute Error (MAE) and Mean Absolute Percentage Error (MAPE). The mathematical representations for these metrics are as follows:

(11) $$\begin{array}{cc} \mathrm{MAE} & =\frac{1}{n\times m}\sum_{i=1}^{n}\sum_{j=1}^{m}\left|Y_{ij}-{\hat{Y}}_{ij}\right|, \end{array}$$

(12) $$\begin{array}{cc} \mathrm{MAPE} & =\frac{100\%}{n\times m}\sum_{i=1}^{n}\sum_{j=1}^{m}\left|\frac{Y_{ij}-{\hat{Y}}_{ij}}{Y_{ij}}\right|. \end{array}$$

In these equations, $Y_{ij}$ and ${\hat{Y}}_{ij}$ denote the actual and predicted temperature values for each element in the 2D temperature field, respectively, with *n* and *m* representing the number of rows and columns in the temperature matrix. The individual errors calculated using these metrics provide insights into the precision at each data point, while the overall error for the validation dataset, obtained by averaging these individual errors, offers an evaluation across the entire dataset.

### 4.1. Performance of Physics-Informed Online Learning

The performance evaluation of the physics-informed (PI) model during the online learning phase involves a structured training procedure. The dataset from each process—B, C, and D—was split such that the first 80% of data were used incrementally as the training set, allowing the model to continuously update and refine its predictions. The remaining 20% of the data serve as the validation set, used to assess the model’s predictive accuracy and to validate its generalization capability on unseen data.

The model’s ability to process new batches of data swiftly is a significant advantage, particularly in real-time applications. On average, updating the model with a new batch of data takes just 0.21 s on Compute Canada’s infrastructure using a single NVIDIA Tesla T4 GPU. This demonstrates the framework’s efficiency and practical utility in scenarios where rapid data processing and immediate decision making are crucial.

**Figure 5:**
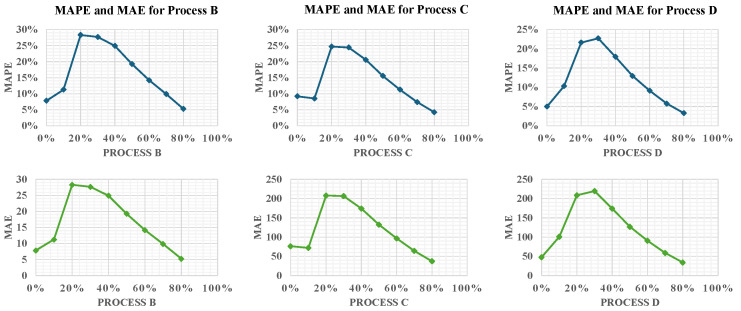
Comparative analysis of MAPE and MAE across processes B, C, and D.

In Figure 5, the MAPE and MAE are illustrated, providing clear visual indicators of the model’s performance across various stages of the learning process. These metrics are plotted against the percentage of process data that were incrementally introduced to the model, which are represented on the x-axis. The y-axis, meanwhile, displays the values of the MAPE or MAE, indicating the model’s error rate at each stage of data integration.

For Process B, the initial spike in the MAPE suggests an adjustment period as the model adapts to changes in process parameters. As more data are processed, a steady decrease in error is observed, highlighting the framework’s capacity to learn and refine its predictions based on closely related baseline conditions. In contrast, Process C, with its introduction of new deposition patterns in addition to varied process parameters, starts with a higher MAPE. This underscores the complexity introduced by the new material properties and deposition strategies. However, the subsequent decline in the MAPE indicates effective model adaptation to these complexities, showcasing its ability to manage multi-faceted changes in AM processes.

Process D shows an initially high MAPE, reflecting the challenge of accommodating a new material and geometric configuration, but this quickly improves as the model assimilates more data and fine-tunes its predictions to the altered geometry. Despite Process B’s closer resemblance to Process A, the modeling for Process D results in lower errors than for Process B. This could be attributed to the circular deposition patterns in Processes A and B, which may complicate thermal management due to surface curvature affecting heat conduction and convection, thereby posing greater modeling challenges than the uniform cubic geometry of Process D. We utilized multi-layer simulations to capture the cumulative thermal effects and inter-layer interactions, which are crucial for accurate temperature predictions and process optimization. However, we recognize the importance of analyzing more complex geometries and their impact on thermal predictions, which will be considered in future work.

### 4.2. Comparison of Proposed Framework with Machine Learning Framework

In this study, we conducted a comparative analysis between the physics-informed (PI) online learning framework and a data-driven approach. As outlined in Section 2.1, the PINN model that is used in the PI framework incorporates three main components: the neural network architecture, physics-informed (PI) inputs, and physics-informed (PI) loss function. In contrast, the data-driven model used for comparison utilizes the same neural architecture but excludes the PI components, focusing solely on data-centric learning methods.

To ensure a comprehensive evaluation, we assessed the performance of both the PI and data-driven frameworks across two critical areas: the entire temperature field of the layer being deposited and specifically within the Heat-Affected Zone (HAZ) and melt pool area. The HAZ is a crucial region in metal AM processes, characterized by the surrounding material of the weld or melt pool that experiences thermal cycling without melting. This zone is drastically influential in microstructural changes due to thermal exposure, which can significantly impact the mechanical properties and integrity of the final product [35]. The accurate identification and management of the HAZ and melt pool are essential for ensuring the quality of manufactured components.

The literature indicates that the identification of the HAZ in AM processes typically involves sophisticated methods such as thermal imaging, metallurgical analysis, and computational modeling. In our study, the HAZ is defined as the region where the temperature exceeds a specific threshold that modifies the microstructure. For materials like 17–4PH stainless steel and Inconel 625, the HAZ temperature thresholds are set at 1050 °C [36] and 960 °C [37], respectively, reflecting their unique thermal characteristics. These established thresholds facilitate the analysis of both the HAZ and melt pool, which is crucial for the comparative evaluation of the frameworks in our study.

**Figure 6:**
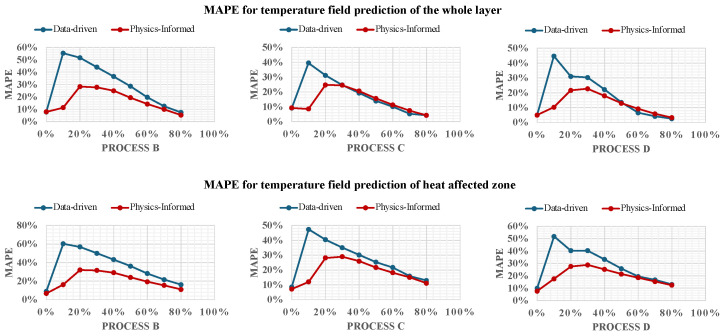
Comparative MAPE of physics-informed and data-driven models across Processes B, C, and D.

Figure 6 presents the MAPEs of both the PI and data-driven frameworks as they predict temperature fields across the entire deposited layer (upper row), the melt pool, and the surrounding HAZ (lower row). In Process B, the PI framework consistently outperforms the data-driven model, exhibiting lower errors when predicting the temperature field for future timestamps as it continuously integrates new data. However, for Processes C and D, despite the PI model initially showing lower error rates—thanks to the incorporation of physics-based knowledge during training—the data-driven model achieves a slightly lower MAPE in the latter half of the process. This shift can be attributed to the additional requirements imposed by the physics-informed constraints in the PI model, which slightly decelerates error reduction.

Regarding the specific areas of the HAZ and melt pool, the PI framework maintains superior performance throughout the online learning process, consistently presenting lower error rates compared to its data-driven counterpart. Notably, the disparity between the PI and data-driven models is more noticeable at the beginning of the online learning phase when less data are available. This underscores the significant advantage of incorporating prior physical knowledge into the neural network, which enhances initial model guidance and prediction accuracy at the early stages. It is important to note that the error rates for both models are generally higher in the HAZ and melt pool areas. This increased error is due to the more complex thermal behavior in these zones, influenced by phenomena such as steep thermal gradients, rapid solidification rates, and varied material properties at high temperatures. These factors complicate the thermal dynamics, making accurate predictions more challenging [20].

**Figure 7:**
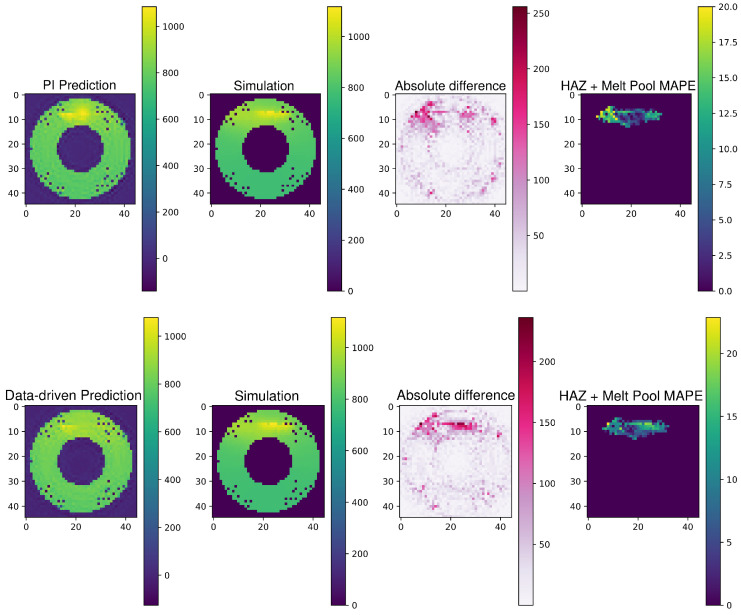
Comparative top view of temperature fields for Process C, showing framework predictions alongside simulation results with physics-informed predictions on top and data-driven predictions below.

The comparative analysis between the PI and the data-driven frameworks is further elucidated by examining their respective outputs against the ground truth provided by simulations. Figure 7 illustrates the predicted temperature fields for both the PINN and data-driven models alongside the simulated “true” temperature distributions for process C.

In the provided examples, both the PI and data-driven frameworks align variably with the simulation results, with most predicted temperatures differing by less than 20 °C from the simulated temperature. Near the melt pool, discrepancies increase, though the PI model generally approximates simulated values more closely, especially around critical areas like the HAZ and melt pool itself. This precision is indicative of the PINN’s ability to incorporate physical laws into its predictions of complex phenomena in metal AM processes.

The figure also displays the absolute difference panels, which quantify the discrepancies between the predictions and the simulations. These discrepancies highlight areas where the models struggle to capture the exact thermal behaviors, potentially due to the intricate dynamics within the melt pool and HAZ that are challenging to model precisely with data-driven approaches alone.

The “HAZ + Melt Pool MAPE” images further provide a focused view of the error distribution within the HAZ, emphasizing the regions where the predictions deviate most significantly from the observed data. This detailed error analysis is critical for refining the models and for understanding the specific conditions under which each model may require further tuning or additional data to enhance the accuracy.

In these comparisons, the PI framework consistently outperforms the data-driven framework, particularly in critical areas such as the HAZ and melt pool, where precise temperature knowledge is crucial for ensuring the quality and integrity of the manufactured parts. This superiority of the PI framework is especially significant when considering that the provided examples are outputs at a stage where only 80% of the data from the new process have been integrated into the models, as indicated in Figure 6. It is noteworthy that the PI framework’s performance advantage becomes even more pronounced when less data are available, underscoring its robust capability to effectively utilize physical laws to predict complex phenomena with limited input data. This makes the PI model particularly valuable in the early stages of new process integration, where data scarcity can often hinder accurate modeling. Additionally, the PI framework’s integration of physical principles allows it to maintain a high prediction accuracy across various conditions and complexities of the AM processes, providing a reliable and efficient tool for process optimization and control. This reliability is crucial for applications where obtaining extensive training data is impractical or time-consuming, thus ensuring consistent quality and performance in real-world manufacturing scenarios.

### 4.3. Effect of Varying Learning Rates

This section evaluates the impact of different learning rate strategies on the performance of the PI framework during its online learning stage. As the model transitions from pretraining on a previous process’s data to incremental data from a new process, selecting an optimal learning rate strategy becomes critical for managing adaptability and accuracy.

We explore three main learning rate strategies: constant, linear increasing, and linear decreasing. A constant learning rate strategy provides a stable update mechanism throughout the learning process, suitable for environments where data properties do not vary significantly. A linear increasing learning rate strategy allows the model to start with cautious adjustments and progressively increase its responsiveness as it adapts to the new data. Conversely, a linear decreasing learning rate strategy enables the model to initially make broad updates and gradually refine these adjustments to focus on detailed patterns.

**Figure 8:**
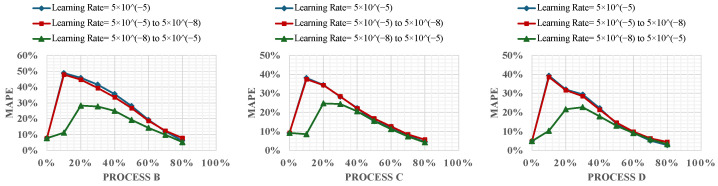
Comparison of learning rate strategies on PINN performance.

Figure 8 illustrates the MAPEs for these strategies, showcasing how each one impacts model accuracy over the training period.

The constant learning rate was set at $5\times 10^{-5}$, a value carried over from the offline learning stage to provide a baseline of stability. In the increasing learning rate strategy, the learning rate began at a much lower value, $5\times 10^{-8}$, intentionally chosen to demonstrate the effect of gradually adapting the learning rate on the model’s performance. This strategy proved particularly beneficial as it allowed the model to adjust more significantly as its confidence in the new data increased, leading to a consistent reduction in error rates throughout the training process. By gradually increasing the learning rate, this approach helps prevent stagnation, ensuring that the model remains dynamic and responsive as it encounters new and increasingly complex data. This calibration of learning rate adjustments fosters a more gradual adaptation to new data, enabling the model to evolve its learning strategy in sync with the unfolding complexities of the process. On the other hand, the decreasing learning rate started at $5\times 10^{-5}$, aiming to quickly assimilate broad patterns before reducing the rate to $5\times 10^{-8}$. However, this approach occasionally resulted in higher error rates in training, indicating that a high initial rate might compromise the model’s ability to adapt to new complexities as they arise.

The analysis indicates that the choice of learning rate strategy and its initial setting plays an important role in the model’s ability to adapt to new data. Among the strategies examined, the increasing learning rate strategy not only facilitated better initial learning with minimal risk of error escalation but also allowed for enhanced adaptability and precision as the complexity of the data increased. This strategy’s success underscores the importance of a dynamic learning rate adjustment in environments where data characteristics are expected to evolve substantially, such as in metal AM.

### 4.4. Effect of Varying Batch Sizes

In this section, we explore the impact of varying the batch sizes of new online data on the performance of the PI online learning framework, specifically focusing on batch sizes of 2, 4, 8, 16, and 32. The batch size is a critical hyperparameter in machine learning that determines the number of training examples used in one iteration to calculate the gradient during the model training process. This parameter significantly influences both the computational efficiency and the convergence behavior of the training algorithm.

**Figure 9:**
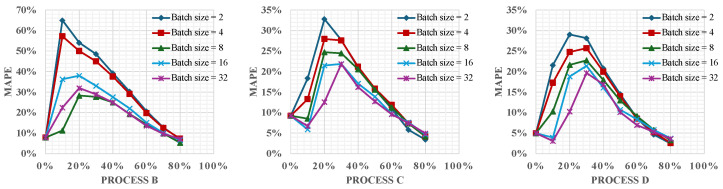
Impact of batch size on model performance across Processes B, C, and D.

Figure 9 illustrates the MAPE for different batch sizes across Processes B, C, and D. This visualization helps assess how the batch size impacts model accuracy and learning dynamics during the online training phase. The results reveal that as the batch size increases, there is a noticeable stabilization in error reduction throughout the online learning process. Larger batch sizes tend to smooth out the learning updates due to the averaging of gradients across more data points. This aggregation diminishes the influence of outliers and reduces the variability of weight updates, leading to a more consistent and gradual decrease in error rates.

Moreover, larger batch sizes offer computational advantages, particularly for real-time applications. Processing larger batches can utilize computational resources more efficiently, potentially speeding up the training process since fewer updates are required per epoch. However, this comes with the caveat that the model must wait for the entire batch of data to be collected and processed before proceeding with an update. This requirement can introduce delays in scenarios where data are being gathered incrementally, such as in real-time monitoring or streaming applications. Hence, there is a trade-off between computational speed and update latency that needs to be carefully managed to optimize real-time performance.

In conclusion, the choice of the batch size is a decision that balances several factors, including error stability, computational efficiency, and responsiveness to new data. Larger batches may be preferable for scenarios where computational speed is crucial and data are abundant, but they require careful consideration of the delay in model updates.

### 4.5. Discussion on Geometry and Multi-Layer Fabrications

We utilized multi-layer simulations with consistent geometries across layers to capture the cumulative thermal effects and inter-layer interactions, which are important for accurate temperature predictions and process optimization. This approach allowed us to systematically investigate thermal behavior and ensure consistency in our predictions.

Geometrical variations, such as changes in the shape and size of the printed part, significantly impact the thermal distribution due to differences in heat conduction, convection, and radiation pathways. Complex geometries can result in non-uniform thermal fields, leading to localized areas of higher or lower temperatures that can affect material properties and structural integrity. The accumulation of heat in certain areas can cause thermal stresses and deformations, making it important to predict these thermal fields accurately. Understanding and predicting these thermal variations are important for refining and optimizing the printing path to ensure uniform temperature distribution, reduce thermal stresses, and improve the overall quality of the final product.

Analyzing more complex geometries and varying geometries across layers is valuable for fully understanding their impact on thermal behavior and inter-layer interactions. This knowledge will enhance the model’s applicability and provide a more comprehensive understanding of metal AM processes.

## 5. Conclusions

In this paper, we introduce the first physics-informed (PI) online learning framework specifically designed for temperature field prediction in metal additive manufacturing (AM), utilizing a physics-informed neural network (PINN). This innovative framework integrates a PINN that includes three main components—a neural network architecture, physics-informed inputs, and physics-informed loss functions. Initially, the PINN is pretrained on a known process during the offline learning stage to establish a foundational model. It then transitions to the online learning stage where it continuously adapts to new, unseen process data by dynamically updating its weights.

Our results demonstrate the robust performance of the PI online learning framework in predicting temperature fields for unseen processes, effectively handling various manufacturing conditions. Particularly notable is its superior performance over data-driven counterparts in predicting temperatures in critical areas such as the Heat-Affected Zone (HAZ) and melt pool. These regions are vital for the overall quality and structural integrity of the manufactured parts, highlighting the importance of precise temperature predictions in these areas. The PINN’s integration of physical laws and prior knowledge provides a distinct advantage, enabling more accurate predictions under diverse conditions. Additionally, our analysis of key operational parameters—including the learning rate and batch size of the online learning process—reveals their roles in optimizing the learning process, further enhancing the framework’s effectiveness.

The key contributions of this study are as follows:

1. Online Learning and Prediction: This study is potentially the first attempt to apply online learning for the real-time modeling and prediction of temperature fields in previously unseen AM processes. This pioneering effort represents an advancement toward adaptable manufacturing technologies.
2. Physics-Informed Integration: We incorporated heat boundary conditions into our framework as the physics-informed loss function, and heat input characteristics as physics-informed input within the neural network. This integration significantly increases the prediction accuracy and reliability.
3. Framework Generality: Our methodology proves highly versatile, demonstrating effectiveness across a diverse range of AM conditions. It can accommodate changes in process parameters, materials, geometries, and deposition patterns, showcasing an essential step toward a universally adaptable AM framework.
4. Improvement in Predictive Accuracy and Process Adaptability: By integrating real-time data with PINNs, this research enhances the predictive accuracy and adaptability of thermal models in metal AM. The framework’s dynamic adaptation to new data and varying conditions ensures precise temperature predictions, improving quality and consistency in AM processes. This advancement over existing methods enables more accurate and reliable thermal modeling, supporting the development of adaptable and efficient AM technologies.

In conclusions, this study represents a pioneering effort in applying physics-informed online learning to metal AM, offering significant improvements in predictive accuracy and operational efficiency. Looking ahead, experimental data from actual metal AM processes can be integrated to enhance the physics-informed machine learning framework. The incorporation of real-world data is expected to improve the accuracy of the model’s predictions by capturing the complex and nuanced behaviors of AM environments. We will also consider more complex geometries and varying geometries across layers in future work to further refine our predictive capabilities.

## Figures and Tables

**Table 1:** Illustrations of four simulated processes. In the geometry and deposition patterns, the solid lines indicate the deposition geometry, and the dashed lines indicate the direction of the laser scanning.

	Process A	Process B	Process C	Process D
**Material**	17-4PH Stainless Steel	17-4PH Stainless Steel	17-4PH Stainless Steel	Inconel 625
**Process Temperature**	2400 °C	2000 °C	2000 °C	2000 °C
**Travel Speed**	10 mm/s	6 mm/s	6 mm/s	20 mm/s
**Number of Layers**	10	10	9	8
**Geometry and Deposition Pattern**	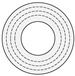	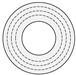	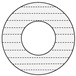	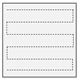

## Data Availability

The original contributions presented in the study are included in the article, further inquiries can be directed to the corresponding author.
